# PBI-05204, a supercritical CO_2_ extract of *Nerium oleander*, suppresses glioblastoma stem cells by inhibiting GRP78 and inducing programmed necroptotic cell death

**DOI:** 10.1016/j.neo.2024.101008

**Published:** 2024-05-31

**Authors:** Sharmistha Chakraborty, Daoyan Wei, Megan Tran, Frederick F Lang, Robert A Newman, Peiying Yang

**Affiliations:** a Department of Palliative, Rehabilitation and Integrative Medicine, The University of Texas MD Anderson Cancer Center, Houston, Texas 77030, United States; b Department of Gastroenterology, Hepatology, and Nutrition, The University of Texas MD Anderson Cancer Center, Houston, Texas 77030, United States; c Department of Neurosurgery, The University of Texas MD Anderson Cancer Center, Houston, Texas 77030, United States; d Phoenix Biotechnology, San Antonio, Texas 78217, United States

**Keywords:** PBI-05204, Glioblastoma stem cells, CD44, GRP78, Necroptosis, Oleandrin

## Abstract

Successful treatment of glioblastoma multiforme (GBM), an aggressive form of primary brain neoplasm, mandates the need to develop new therapeutic strategies. In this study, we investigated the potential of PBI-05204 in targeting GBM stem cells (GSCs) and the underlying mechanisms. Treatment with PBI-05204 significantly reduced both the number and size of tumor spheres derived from patient-derived GSCs (GBM9, GSC28 and TS543), and suppressed the tumorigenesis of GBM9 xenografts. Moreover, PBI-05204 treatment led to a significant decrease in the expression of CD44 and NANOG, crucial markers of progenitor stem cells, in GBM9 and GSC28 GSCs. This treatment also down-regulated GRP78 expression in both GSC types. Knocking down GRP78 expression through GRP78 siRNA transfection in GBM9 and GSC28 GSCs also resulted in reduced spheroid size and CD44 expression. Combining PBI-05204 with GRP78 siRNA further decreased spheroid numbers compared to GRP78 siRNA treatment alone. PBI-05204 treatment led to increased expression of pRIP1K and pRIP3K, along with enhanced binding of RIPK1/RIPK3 in GBM9 and GSC28 cells, resembling the effects observed in GRP78-silenced GSCs, suggesting that PBI-05204 induced necroptosis in these cells. Furthermore, oleandrin, a principle active cardiac glycoside component of PBI-05204, showed the ability to inhibit the self-renewal capacity in GSCs. These findings highlight the potential of PBI-05204 as a promising candidate for the development of novel therapies that target GBM stem cells.

## Introduction

Glioblastoma constitutes a predominantly heterogeneous population of aggressive and invasive primary malignant brain tumors among human adults. Classified as a grade IV astrocytoma by the World Health Organization (WHO)[1,2], glioblastoma (GBM) carries a grim prognosis with an overall survival of only 10-20 months[3]. GBM is characterized by extensive hypoxia, angiogenesis, proliferation, and invasion[4]. Standard treatment practices such as surgical resection, radiotherapy, and chemotherapy with temozolomide (TMZ) have increased median patient survival in adults, but development of resistance to these therapeutic measures often results in GBM tumor regrowth and patient mortality[5]. The resistant cancer cells often display stem cell-like properties[[6], [7], [8]]. Unfortunately, efforts to identify or target these glioblastoma stem cells (GSCs) have mostly been proven to be ineffective. Thus, there is an unmet need for the development of novel effective therapeutic strategies to specifically target GSCs to improve the survival of patients with GBM.

Glioblastoma stem cells isolated from human tumors have the ability to develop tumors when transplanted into nude mice. These cells have been shown to be one of the primary causes of tumor proliferation, maintenance, malignant recurrence, and metastasis in GBM due to their self-renewal, multilineage differentiation, and proliferation properties[8]. GSCs assist in development of modified tumor microenvironments through multifaceted crosstalk within their niche[9]. Establishing efficient models in vitro and targeting these in vivo to exploit resistant cell populations, like GSCs, is important in identifying innovative treatment strategies which will synergize with existing standard of care modalities.

Emerging evidence has shown that certain cardiac glycosides, such as oleandrin, can reduce the growth of various types of tumors including GBM and have a strong potential for use as novel cancer therapeutic agents[[10], [11], [12]]. Using a systemic biology-based drug repositioning screen, several cardiac glycosides were recently identified among 1300 candidate molecules as potent inhibitors for the growth of group 3 and 4 medulloblastomas[11]. For example, proscillaridin A, a cardiac glycoside, was shown to inhibit the proliferation of GBM cells and GSCs by induction of G2/M phase cell cycle arrest[13] and digoxin has been shown to suppress the stemness of human glioblastoma cells by inhibiting HIF-1α[14]. We have recently reported that PBI-05204, a supercritical CO_2_ extract of *Nerium oleander*, strongly inhibits the growth of glioblastoma cells and their xenograft and orthotopic models by induction of apoptosis and suppression of the PI3K/mTOR pathway and the stemness of these cells[15]. However, how PBI-05204 regulates the stemness of glioblastoma cells has yet to be determined.

In the present study, we examined the anti-stemness potential of PBI-05204 in patient derived glioblastoma stem cells and explored underlying molecular mechanisms. We report that PBI-05204 has profound anti-stemness effects in GSCs and anti-proliferative activity in its xenograft GSC model by downregulation of GRP78 and induction of programmed necroptotic cell death.

## Materials and methods

### Cell lines and culture

Glioblastoma stem cells (GSC)s were isolated and characterized from patient derived GBM tumor tissues. The following cell lines MDA-GSC20 (GSC20), MDA-GSC28 (GSC28), were obtained from Dr. Frederick F Lang, Brain Tumor Center, The University of Texas MD Anderson Cancer Center, Houston TX. GBM9 was a gift from Dr. Amyn A Habib, UT Southwestern Medical Center, Dallas, TX. TS543 was obtained from Dr. Krishna PL Bhat in the Department of Translational Medicine at the University of Texas MD Anderson Cancer Center. The GSC cell lines were cultured in DMEM/F-12 medium (Corning, Durham, NC) with neuronal cell culture Gibco B-27 supplement minus Vitamin A (Thermo Fisher Scientific, San Francisco, CA), recombinant human basic fibroblast growth factor (bFGF, 20 ng/ml), and recombinant human epidermal growth factor [(EGF, 20 ng/ml) STEMCELL Technologies, Vancouver, Canada] in Corning low attachment (ULA) plates/flasks as per manufacturer's instructions at 37°C in the presence of 5 % CO_2_. Accutase, a cell dissociation solution (Sigma-Aldrich, St. Louis, MO), was used to dissociate spheres for passaging and experimental setup.

### Drugs and treatment

PBI-05204 is a supercritical CO_2_ extract of *Nerium oleander* leaves characterized using an AccuTOF-DART mass spectrometer (Jeol UAS, Peabody, MA). Specific content of the extract has previously been reported[15,16]. PBI-05204 was dissolved in DMSO and then further diluted with complete GSC culture medium prior to use. Oleandrin was obtained from ChromaDex (Longmont, CO).

### Sphere formation and sphere viability assays

GSC spheres were dissociated into single cells with Accutase and counted. Single cells (500/well) were plated in 96 well low attachment plates in complete GSC medium and cultured at 37°C under 5 % CO_2_ in the presence or absence of PBI-05204. Spheres were counted using an EVOS-FL auto imaging system (Invitrogen, Waltham, MA). Sphere diameters were measured using ImageJ software. GSC sphere viability was measured using Cell Titer Glo 3D cell viability assay reagent (Promega, Madison, WI) as per the manufacturer's protocol.

### Xenograft mouse model

The animal care protocol was approved by the Animal Care and Use Committee at the University of Texas MD Anderson Cancer Center. GBM9 cells were cultured in GSC culture medium as previously described until mature spheres were formed[17]. Following dissociation, cells were counted and strained through filters to achieve single cell suspensions. Cells were counted in an automated cell counter (Bio-Rad, Hercules, CA) and were resuspended in complete GSC culture medium prior to injection (1.0×10^6^ per 100μl of medium) into the flanks of female athymic mice (6-8 weeks old). PBI-05204 treatment was administered by oral gavage daily at two different dose levels, 10 mg/Kg and 20 mg/Kg for three weeks. The body weights of mice were measured weekly. Tumor diameters were measured with a digital caliper, and the tumor volume in mm^3^ was determined by the formula: Volume = (width)^2^ x length/2 where width is shorter than length. At termination of the study, mice were euthanized, and blood was collected by cardiac puncture. Tumor tissues were collected and either flash frozen or fixed in 10 % formalin for further analysis.

### Immunoprecipitation and Western blot

GSC cell lysates were prepared in ice cold lysis buffer (50 mM Tris-HCl, pH 7.4, 200 mM sodium chloride, 2 mM EDTA, 0.5 % Na-deoxycholate, 1 % NP-40) in the presence of protease inhibitors, aprotinin (0.5 mg/ml), leupeptin (0.5 mg/ml), PMSF (1 mM) and phosphatase inhibitors with sodium orthovanadate (0.2 mM) and sodium fluoride (50 mM) being added freshly. For immunoprecipitation, cell lysates were incubated with the indicated antibodies (2 µg) at 4°C overnight on a rotating platform. Protein A/G plus agarose beads (Santa Cruz Biotechnology, Inc., Dallas, TX) were added the following day and incubated at 4° C for an hour. The beads were then washed 3-4 times with NETN buffer (200 mM Tris-HCl, pH 7.8, 100 mM NaCl, 1 mM EDTA, 10 % glycerol, 0.1 % NP-40 and protease inhibitors) and samples were subjected to SDS-PAGE. Western blot analysis was performed using indicated antibodies and visualized using a previously described method[18].

### Transmission electron microscopy (TEM)

Transmission electron microscopy was used for detection of fine details of organelle structure within GSCs and was performed in the High-Resolution Electron Microscopy Facility at MD Anderson Cancer Center. GBM9 and GSC28 cells were plated in equal numbers in 12 well plates (low binding). The GSC spheres were treated with PBI-05204 using one of three different concentrations for 24 hr and were then washed with PBS (without Ca^++^/Mg^++^) prior to treatment with EM fixative containing 3 % glutaraldehyde and 2 % paraformaldehyde in 0.1 M cacodylate buffer (pH 7.3). They were then washed in 0.1M sodium cacodylate buffer and treated with 0.1 % Millipore-filtered cacodylate buffered tannic acid, post-fixed with 1 % buffered osmium and stained with 0.1 % Millipore-filtered uranyl acetate prior to dehydration with increasing concentrations of ethanol followed by infiltration and embedding in LX-112 medium. The samples were then polymerized in a 60ºC oven for approximately three days. Ultrathin sections were cut using a Leica Ultracut microtome (Leica, Deerfield, IL) and followed by staining with uranyl acetate and lead citrate in a Leica EM Stainer. The samples were examined in a JEM 1010 transmission electron microscope (JEOL USA, Inc., Peabody, MA) using an accelerating voltage of 80 kV. Digital images were obtained using an AMT imaging system (Advanced Microscopy Techniques Corp., Danvers, MA).

## Results

### PBI-05204 inhibits the growth and self-renewal capacity of patient derived glioblastoma stem cells in vitro

**Fig. 1:**
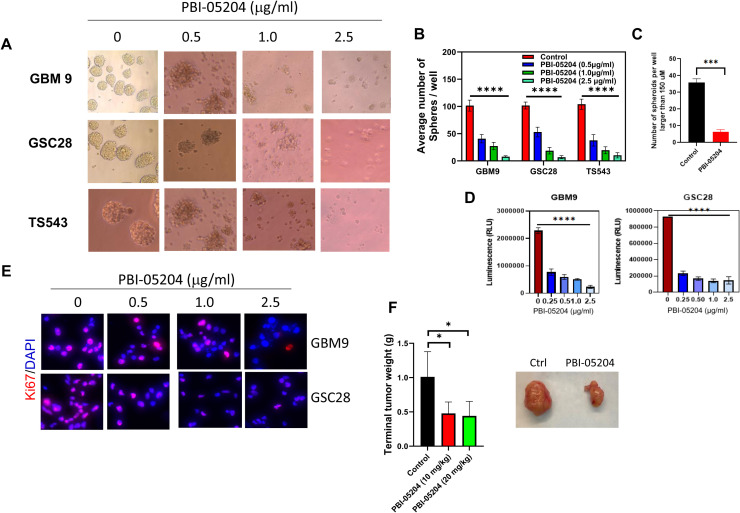
**PBI-05204 inhibits GSC self-renewal capacity and the growth of GSC-derived xenograft tumors. A.** Spheroid formation of GSCs, GBM9, GCS28, and TS543 cells, treated with PBI-05204 for 72 hr. Pictures were taken at 20×. **B.** Quantitative analysis showed that PBI-05204 administration dose-dependently reduced the number of GBM9, GSC28 and TS543 cells. **C.** Number of GBM9 spheres larger than 150 µM treated with PBI-05204 for 72 hr. **D.** Proliferation of GBM9 and GSC28 cells after exposure to PBI-05204 at indicated concentrations measured by Celltiter Glo assays. **E.** Immunofluorescence staining of Ki67 in PBI-05204 treated GBM9 and GSC28 cells. Pink represents staining of Ki67 positive cells. **F.** Terminal tumor weight of GBM9 mouse xenografts after treatment of mice with oral gavage of PBI-05204 at indicated doses for 3 weeks (n = 6). Data are presented as mean ± SD. *p < 0.05, ***p < 0.001; **** p < 0.0001 versus control.

Glioblastoma stem cells grown as non-adherent spheres have been shown to represent a reliable *in vitro* assay that can be used to assess their self-renewal ability in different GSC models[[19], [20], [21]]. The self-renewal capacities of three different patient derived cell lines, GBM9, GSC28, and TS543 were evaluated in the presence of PBI-05204 for 48h in 96 well plates. As shown in Fig. 1A, there are decreases both in tumor sphere number and size that are dependent on the concentration of PBI-05204. Morphologically, PBI-05204 treatment led to necrotic cell death compared to that of control in GSCs (Fig. 1A). GSC sphere count showed a significant decrease in cell survival due to the presence of PBI-05204 even at a low concentration (0.5 μg/ml) (Fig. 1B). GSC sphere diameters were calculated using ImageJ and demonstrated a significant reduction of GSC spheres larger than 150 μm after treatment with PBI-05204 (Fig. 1C). Further, sphere/cell viability was assessed using a 3D cell viability luminescent based assay. As shown in Fig. 1D, there was a significant reduction in cell viability in the presence of PBI-05204 at even the lowest concentration (0.25 μg/ml) for both GBM9 and GSC28 cells. The relative expression of cell proliferation marker Ki67 was assessed by immunofluorescence staining. There was a clear reduction of Ki67 expression upon PBI-05204 treatment in a concentration-dependent manner (Fig. 1E) in both GBM9 and GSC28 cells. To evaluate the role of PBI-05204 with respect to *in vivo* tumor formation, a GBM9 mouse xenograft model was used. Patient derived GBM9 GSCs were injected into the flanks of athymic mice. When the subcutaneous tumors were visible after one week, mice were randomized into three groups: Control, and PBI-05204 at 10 mg/Kg or 20 mg/Kg. Mice were treated with PBI-05204 or control vehicle by oral gavage daily for 3 weeks. There was a significant reduction in tumor weight and size in both 10 mg/Kg and 20 mg/kg PBI-05204 treated groups compared to that of vehicle control (Fig. 1F).

### PBI-05204 treatment suppresses the expression of stem cell markers

**Fig. 2:**
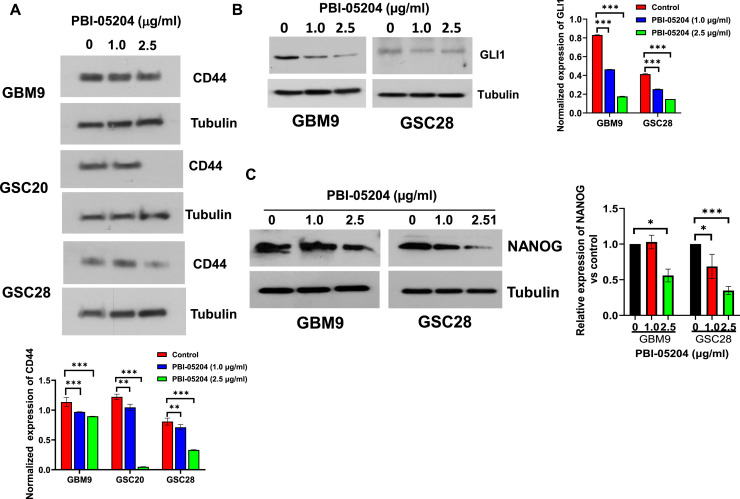
**PBI-05204 down-regulates stem cell markers in GSCs.** GSCs were plated 48 hr and then were treated with PBI-05204 at indicated concentrations for 24 hr prior to determination of protein expression of stem cell makers by western blot analysis. A. CD44 expression in GBM9, GSC20, and GSC28 GSCs. B. GLI1 expression in GBM9 and GSC28 GSCs. C. Western blot of NANOG protein in GBM9 and GSC28 GSCs. Data are presented as mean ± SD. * p < 0.05, ** p < 0.01; ***p < 0.001; **** p < 0.0001 versus control.

An important goal of GBM therapy is successful targeting of GSCs, a subpopulation of cells that contribute to therapy resistance and tumor relapse. The expression of various stem cell markers in GSCs contributes to a better understanding of the fundamental mechanisms in glioma tumorigenesis[19,20]. The transmembrane protein CD44 plays a prominent role in tumor development[22]. In the present study PBI-05204 significantly suppressed the expression of surface marker CD44 in a concentration-dependent manner in patient derived GBM9, GSC20 and GSC28 cell lines (p < 0.01, Fig. 2A). Overexpression of SOX2 mRNA has also been demonstrated to be present in gliomas although SOX2 expression in adult brain is almost undetectable. SOX2 silencing in GSCs has previously been shown to result in a loss of tumorigenicity[23,24]. Our data show that there was a PBI-05204 mediated inhibition of SOX2 protein in TS543 (Figure S1A) and GSC28 (data not shown) cell lines after 24h treatment with PBI-05204. There was no detectable SOX2 expression in the GBM9 cell line (Figure S1A). Another important GSC stem cell marker is NANOG, a heterodomain protein which is involved in the maintenance of pluripotency of embryonic stem cells (ESCs) and is highly expressed in human blastocyst's inner cell mass[25]. Expression of NANOG in gliomas has been previously demonstrated in GSCs[[26], [27], [28]]. Our data show that PBI-05204 markedly blocks GLI1 activation (p < 0.01, Fig. 2B) and significantly decreases NANOG expression in both GBM9 and GSC28 cells, respectively (Fig. 2C).

### PBI-05204 treatment decreases GRP78 protein expression in GSCs

**Fig. 3:**
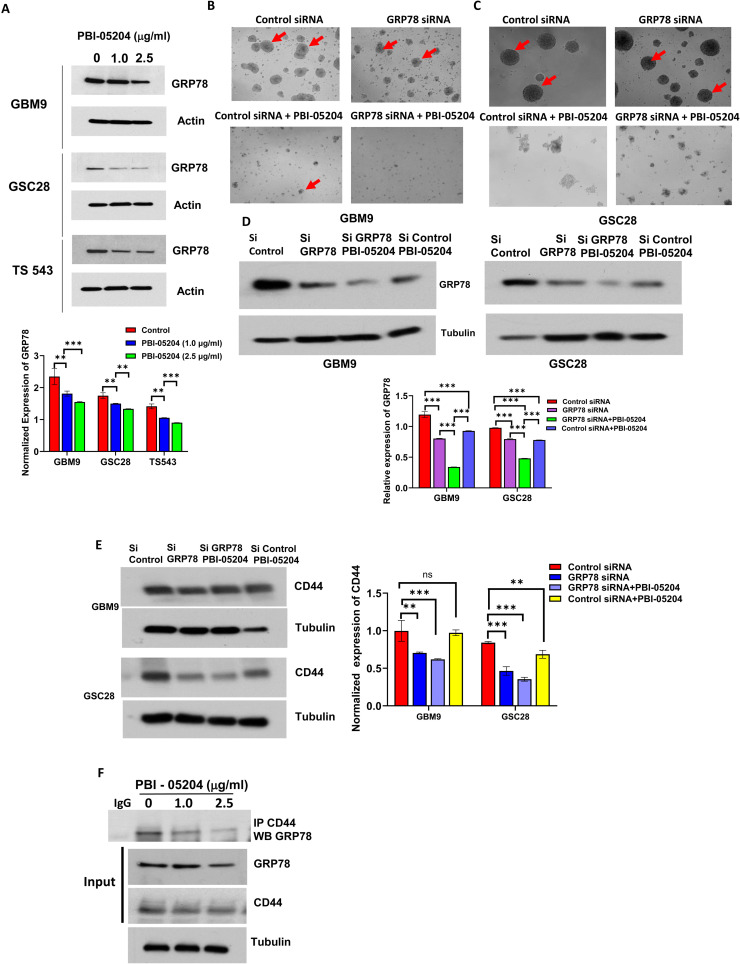
**Reduced GRP78 expression enhances the anti-stemness potential of PBI-05204 via CD44.** A. Western blot analysis of GRP78 protein in PBI-05204 treated GSCs. **B&C.** Spheroid formation in control siRNA or GRP78 siRNA transfected GBM9 (B) and GSC28 (C) GSCs treated with vehicle control or PBI-05204 (1.0 μg/ml) for 24 hr. Red arrows point to the spheroids. **D**. Expression of GRP78 protein in control siRNA or GRP78 siRNA transfected GBM9 and GSC28 GSCs treated with vehicle control or PBI-05204 (1.0 μg/ml). **E**. Expression of CD44 in control siRNA transfected or GRP78 silenced GBM9 and GSC28 GSCs treated with vehicle control or PBI-05204 (1.0 μg/ml). **F**. Immunoprecipitation assay showing deceased interaction of GRP78 protein with CD44 protein in PBI-05204 treated GBM9 cells. Data are presented as mean ± SD. ** p < 0.01; ***p < 0.001.

GRP78, an important ER chaperon, functions as a vital component in many cellular processes such as targeting misfolded proteins for proteasomal degradation and regulation of calcium homeostasis. It is expressed in high levels in diverse cancer types such as lung, breast, and colon cancer and in highly malignant and invasive glioblastoma[29,30] . As shown in Fig. 3A, the data demonstrate a significant inhibition of GRP78 expression by PBI-05204 in a concentration-dependent manner in GBM9, GSC28 and TS543 cells (p < 0.01). To examine the effect of GRP78 silencing on self-renewal and growth in GSCs, siRNA was used in both GBM9 and GSC28 cells. While GRP78 silencing led to a moderate inhibition of self-renewal capacity of GBM9 and GSC28 cells, the data demonstrate a combined effect of PBI-05204 in addition to GRP78 silencing with respect to a reduction of stemness of GSCs (Fig. 3B, C) as denoted by both reduced spheroid numbers as well as size. Western blot analysis showed that while the GRP78 protein expression in GRP78 silenced GSCs was moderately and significantly lower than of that control siRNA transfected GBM9 and GSC28, a combination of siGRP78 and PBI-05204 led to a greater and significant reduction in GRP78 expression compared to that of cells transfected with GRP78 siRNA alone or those treated only with PBI-05204 (Fig. 3D).

To understand the effect of GRP78 silencing alone or in combination with PBI-05204 on established stem cell markers, the expression of CD44 in GRP78 silenced cells with or without PBI-05204 was determined. CD44 protein expression was lower in GRP78 silenced GBM9 and GSC28 cells with an even greater reduction in CD44 expression in PBI-05204 treated GRP78 silenced cells compared to that of control. This was especially pronounced in GSC28 cells (Fig. 3E). A GRP78 interacting partner is a splice variant of CD44v which was discussed by Tseng CC et al[31] in tamoxifen resistant breast cancer. The ability of PBI-05204 to inhibit GSC stemness via a disruption of GRP78 binding to CD44 was noted as shown by endogenous immunoprecipitation (Fig. 3F).

### PBI-05204 activates a necroptotic cell death pathway

**Fig. 4:**
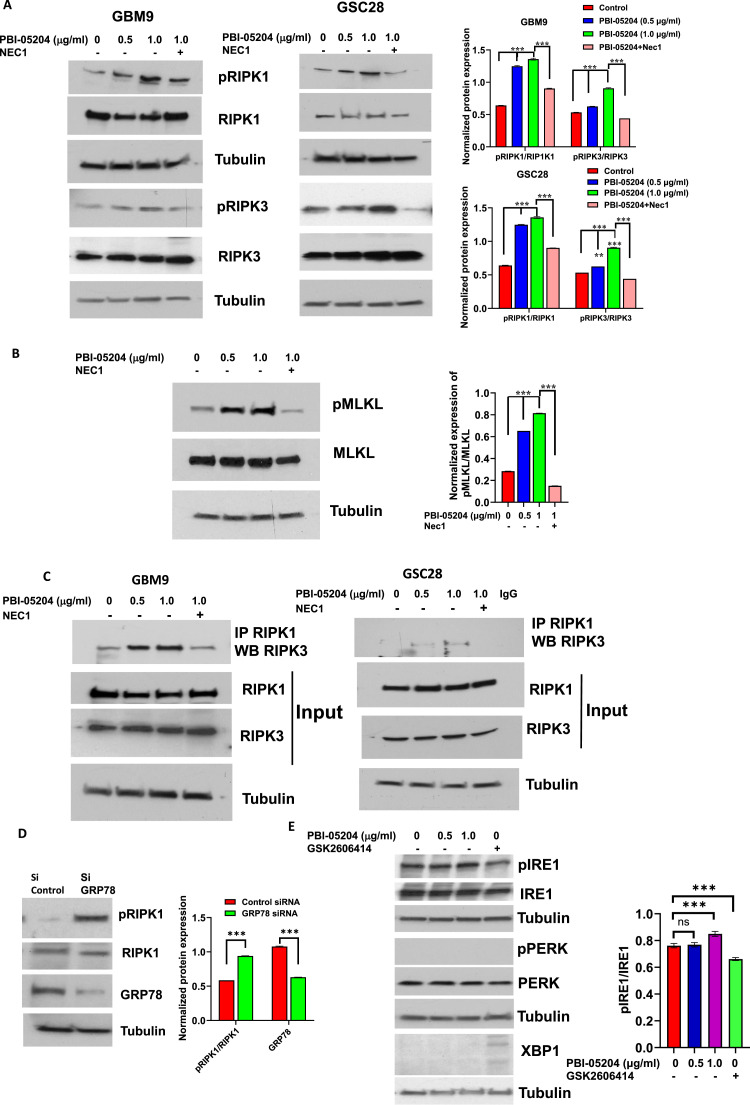
**PBI-05204 treatment led to necroptotic cell death via downregulation of GRP78 in GSCs. A.** Protein expression of necroptosis markers RIPK1, pRIPK1, RIPK3, and pRIPK3 in GBM9 (**left**) and GSC28 (**right**) cells after exposure to PBI-05204 at indicated concentrations with or without RIPK1 inhibitor NEC1. **B.** Protein expression of pMLKL and MLKL in GBM-9 cells after being treated with PBI-05204**. C.** The binding of RIPK1-RIPK3 by endogenous immunoprecipitation. GBM9 (left) or GSC28 (right**)** cells were treated with PBI-05204 at indicated concentrations with or without NEC1 for 24 hr and then immunoprecipitated with RIPK1 antibody followed by an examination of protein expression in RIPK1 IP cells by western blot analysis. **D**. Protein expression of RIPK1 and pRIPK1 in GRP78 silenced GBM9 cells. E. Protein expression of ER stress markers pIRE1, IRE1, pPERK, PERK, and XBP1 splicing in GBM9 cells after being treated with PBI-05204 (0.5 and 1.0 μg/ml) and GSK2606414 (10 μM) for 20 hr. Data are presented as mean ± SD. ** p < 0.01; ***p < 0.001.

The effects of PBI-05204 on changes in GSC morphology (necrotic) and cell death (3D cell viability assay) were observed in a sphere formation assay (Fig. 1A), suggesting the involvement of programmed cell death pathways upon treatment with PBI-05204 in GSCs. Recent studies have shown that necroptosis is an unfavorable prognostic marker of GBM[32]. To investigate whether this pathway is involved in PBI-05204 mediated cell death, the central core of necroptotic pathway components was examined. This involves the activities of phosphorylation-driven activation of RIPK1 and RIPK3 and MLKL[33]. RIPK3 is critical for triggering RIP1 kinase that regulates RIP1-dependent “necrosome” formation[34]. The data show a significant increase in RIPK1 and RIPK3 phosphorylation in the presence of PBI-05204 in a concentration dependent manner in GBM9 and GSC28 cells (p < 0.001) Fig. 4A). When GSC cells were treated with both PBI-05204 and RIPK1 inhibitor Necrostatin1 (NEC1), the levels of phosphorylated RIPK1 and RIPK3 in PBI-05204 and NEC1 treated GSC were significantly less than that of PBI-05204 only treated group (p < 0.001, Fig. 4A). RIPK1 binds to RIPK3 via the RIPK homotypic interaction motif (RHIM) at the C-terminal end to form the RIPK1-RIPK3 complex which is essential in the activation of the necroptosis pathway[33]. The downstream signaling protein MLKL is phosphorylated which then leads to oligomerization of MLKL which, in turn, disrupts the integrity of the plasma membrane and the intracellular membrane, characteristics of necroptosis[35]. Indeed, as shown in Fig. 4B, PBI-05204 treatment led to upregulation of pMLKL while NCE1 blocked elevated pMLKL in GBM9 cells (p < 0.001). Binding of RIPK1 and RIPK3 was examined by endogenous immunoprecipitation. An increase in RIPK1-RIPK3 binding was noted in the presence of PBI-05204 in both GBM9 and GSC28 cell lines (Fig. 4C). Furthermore, RIPK1-RIPK3 binding was reduced in the presence of the RIPK1 inhibitor NEC1 (Fig. 4C). In light of GRP78 silencing triggering GSC cell death as shown in Fig. 3B, we investigated the potential cell death pathway that was involved and found that RIPK1 phosphorylation was significantly increased in GRP78 silenced GBM9 cells (p< 0.001, Fig. 4D). Considering that GRP78 is important in controlling UPR and ER stress responses, we then examined unfolded protein response pathway upon PBI-05204 treatment in GSCs. While GSK2606414, a known PERK inhibitor also inhibits IRE1[36], significantly reduced pIRE1/IRE1 in GBM9 cells compared to that of control cells, the results showed a minimum change of pIRE1/IRE1 in GBM9 cells treated with the lower concentration of PBI-05204 (0.5 μg/ml), though a moderate and significant increase in the ratio of pIRE1 over IRE1 in higher concentrations of PBI-05204 treated GBM9 GSCs (Fig. 4E). However, the protein expression of pPERK was not detectable in GBM9 cells regardless of the treatment. Similarly, no XBP1 splicing, the downstream effector of IRE1 response, was noticeable in GBM9 cells (Fig. 4E), suggesting PBI-05204 induced GRP78 changes might not be associated with UPR and ER stress response.

### PBI-05204 mediated changes in cell organelle structures

**Fig. 5:**
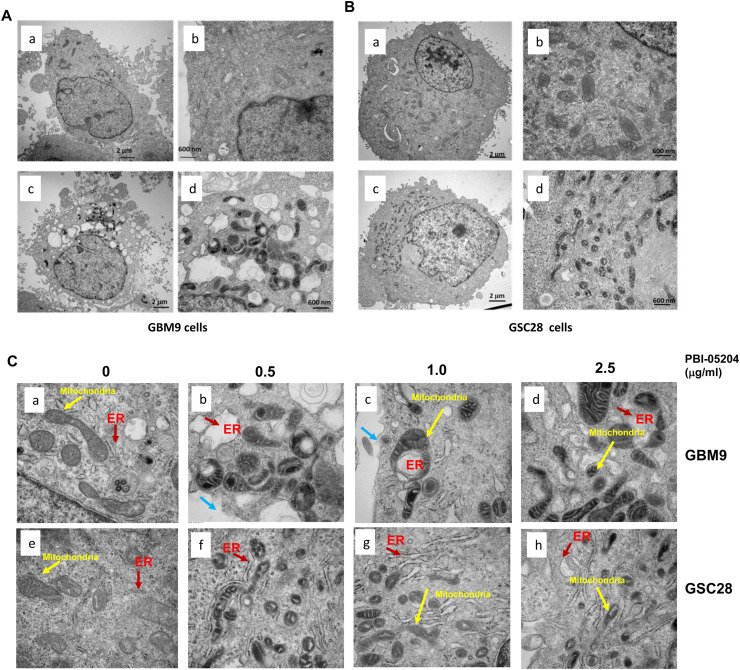
**Transmission electron micrographs show morphologic changes in GSCs treated with PBI-05204. A,** TEM images of GBM9 cells treated with control vehicle (a&c) or PBI-05204 (0.5 ug/ml, b&d) for 24 hr. B. TEM images of GSC28 cells treated with control vehicle (a&c) or PBI-05204 (0.5 ug/ml, b&d) for 24 hr. Note the normal appearance and location of mitochondria as well as normal endoplasmic reticulum (ER) throughout the cytoplasm (a&c). Images a&c were taken at 7500x; Images b&d were taken at 25,000x. **C.** TEM images of GBM9 and GSC28 cells treated with PBI-05204 at indicated concentrations for 24 hr showing condensed mitochondria (yellow arrows), swollen ER (red arrows) and damaged cell membranes (blue arrows). Images were taken at 50,000x.

Cell organelles exist in highly dynamic cellular networks that communicate with each other to carry out fundamental functions. Endoplasmic reticulum (ER) is the primary site for protein biosynthesis and secretion as well as protein folding. The ER plays an important role in lipid biosynthesis, metabolism, calcium storage and signaling[37]. The ultrastructural changes in cellular organelles in PBI-05204 treated GSCs were examined using Transmission Electron microscopy (TEM). PBI-05204 treatment led to ultrastructural changes that were associated with alterations of mitochondria and ER morphology (Fig. 5). Even at a low concentration of 0.5 µg/ml, PBI-05204 induced extensive dilation of ER appearing as cytosolic double membrane vacuoles in both GBM9 and GSC28 cells. The swollen ER morphology was especially prominent in GBM9 cells (Fig. 5A, B) as noted by actual disruption of ER integrity. Mitochondrial morphology changes during programmed cell death result in small, round, and more numerous organelles[[38], [39], [40], [41], [42]]. As shown in Fig. 5C, mitochondrial cristae were disordered and rounder in PBI-05204 treated GSCs cells compared to controls. PBI-05204 treatment also resulted in a progressive reduction in the number of normal ellipsoidal mitochondria and elongation of their profile that was accompanied by a clearing of the inner matrix compartment (Fig. 5C, b-d & f-h) compared to that of the vehicle control group (Fig. 5C-a&e). Broken cell membranes were also observed in GBM9 cells (Fig. 5C-c), indicating the occurrence of necroptosis-mediated programmed cell death.

### Oleandrin inhibits the stemness of GSCs and downregulates stem cell markers

**Fig. 6:**
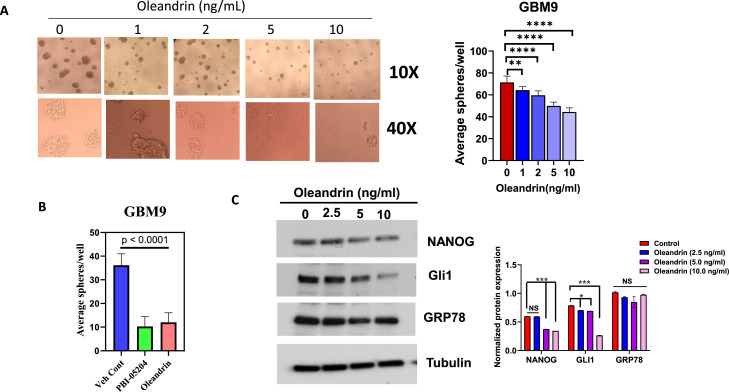
**Oleandrin treatment reduced the stemness of GCSs. A.** Spheroid formation of GBM9 cells after exposure to oleandrin at indicated concentrations for 72 hr. B**.** The average number of spheroids formed from GBM9 cells treated with PBI-05204 provided the equivalent levels of oleandrin as samples treated with oleandrin. **C.** Western blot analysis of stem cell markers and GRP78 in oleandrin-treated GBM9 cells. Data are presented as mean ± SD. * p < 0.05; ** p < 0.01; *** p < 0.001; **** p < 0.0001 versus control.

The antitumor activity of oleandrin, a bioactive compound of PBI-05204, has been well documented by other investigators as well as our own research[[43], [44], [45]]. However, whether oleandrin can impact cancer stem cells, especially on patient derived glioblastoma stem cells, has not previously been evaluated. To identify potential compounds responsible for PBI-05204-elicited anti-stemness activity in GSCs, the effect of oleandrin on stemness and stem cell markers of GSCs was examined. Similar to PBI-05204 treated GSCs, oleandrin-treated GBM9 cells exhibited significantly fewer spheroids compared to the vehicle control. Furthermore, the anti-stemness of oleandrin in GBM9 cells was concentration-dependent (Fig. 6A). Interestingly, upon treatment of GBM9 cells with PBI-05204 or oleandrin at an equivalent concentration to that in PBI-05204 (1 μg/ml), both groups exhibited a decreased number of spheroids compared to the vehicle control GBM9 cells (Fig. 6B). In contrast, when TS543 GSCs were treated with oleandrin and PBI-05204 containing a similar amount of oleandrin, PBI-05204 treated TS543 GSCs had 67 % fewer spheroids than that of vehicle control treated GSCs whereas oleandrin led to about a 34 % reduction in spheroid formation (Supplement Figure S2). The result of western blot analysis showed that oleandrin treatment led to downregulation of NANOG and GLI1 protein expression in a concentration dependent manner in GBM9 cells (Fig. 6C). The reduction of GRP78 protein was also observed in oleandrin treated GBM9 cells but was less obvious than that produced by PBI-05204 treatment (Fig. 3A). These data suggest that oleandrin is an important bioactive component contributing to PBI-05204′s anti-stemness in GSC cells.

## Discussion

Glioma stem cells have recently been recognized as ‘seeds’ driving tumor growth and recurrence, making them promising targets for innovative therapeutic development. Hence, there is an urgent need to better understand and evaluate the efficacy of agents such as PBI-05204 against GSCs. The importance of targeting Na,K-ATPase as a means of controlling GBM growth and proliferation has previously been noted[46]. Various cardiac glycosides that specifically target this enzyme have been reported to interrupt key intracellular pathways suggesting that this is an effective means of attacking GBM cells. Specifically, cardiac glycoside compounds such as digoxin, ouabain[47], proscillaridin A[13,48], bufalin[49], and lanatoside C[50,51] have been reported to suppress GBM growth in vitro and in vivo. Our recent research has shown that PBI-05204 as well as a key cardiac glycoside component, oleandrin, can also inhibit the growth of human glioblastoma. This botanical drug acts in a synergistic manner with radiotherapy[15] and has been shown to modulate key GSC renewal properties[52] . These include 1) Notch signaling which, once aberrantly activated, contributes to cancer progression[53,54], 2) the Hedgehog signaling pathway that exerts its biological effects through a signaling cascade that culminates in a change of balance between activator and repressor forms of glioma-associated oncogene (Gli) transcription factors[55,56], 3) WNT/β-catenin signaling which has been shown to regulate stem cell renewal[57,58] and NF-kβ signaling family of transcription factors that plays an essential role as stressors in the cellular environment, and controls the expression of important regulatory genes such as immunity, inflammation, death, and cell proliferation[[59], [60], [61]]. While the ability of cardiac glycoside compounds to interfere with these key pathways is important, there remains a need to better understand the potential of oleander extracts and key component molecules like oleandrin to specifically affect GSC proliferation and survival.

The expression of various stem cell surface (CD133, CD44, CD15) and intracellular stem cell markers (Nestin, SOX2) are correlated with cancer stem cell features, such as self-renewal and tumor initiation[19,20,23]. The stem cell markers discovered in GBM by Singh et al.[19,62] suggest that the expression of these in GSCs contributes to the development and progression of glioma tumorigenesis. Reports have shown, for example, that high expression of CD44 is associated with poor prognosis in patients with GBM and is an important marker for the aggressive mesenchymal type GSCs[8,63]. CD44 promotes GBM aggressiveness by increasing tumor cell invasion, proliferation and resistance to standard chemoradiation therapy[22]. We have demonstrated that glioblastoma stem cell markers like CD44 and the stemness-inducing reprogramming transcription factor SOX2 are downregulated in GSCs after PBI-05204 treatment which was consistent with our previous published study[52].

Another embryonic stem cell factor NANOG, is a homeodomain protein involved in the maintenance of embryonic stemness and pluripotency and is expressed widely in adult human tissues including human brain[64,65]. Zbinden M et al[25] have shown that NANOG modulates GSCs clonogenicity, and helps in maintenance of GSC proliferation, pluripotency, and is regulated by Sonic Hedgehog-Gli1 signaling. It has been reported previously that NANOG regulates human glioma growth by maintaining stemness[25,66,67] and disruption of the (SHH)-GLI-NANOG network is important for targeting glioblastoma stem cells[68]. In the present study, we demonstrate for the first time that PBI-05204 inhibits Gli1 activation which may be responsible for inhibition of NANOG. However, details as to how PBI-05204 affects SHH-GLi1 signaling will require future research.

Normal brain tissues have a lower expression of GRP78 than other normal tissues. However, among cancer tissues, glioblastoma has a very high expression of GRP78[69]. Of importance, GRP78 expression has been reported to increase with the pathologic grade of glioma[70] and is considered strongly associated with glioma progression. Few studies to date have investigated the relative expression of GRP78 in human GSCs. Our data suggests that GRP78 is an important factor for the maintenance of stemness and self-renewal capacity in GSC cell lines which have been inhibited by PBI-05204. This was confirmed by GRP78 siRNA transfection experiments as shown in Fig. 3B&C. Our data demonstrate that the lower the expression of GRP78 (as shown in GRP78 silencing plus PBI-05204 treated GSC cells compared to treatment with either approach alone), the less self-renewal capacity and greater cell death occur in the co-treated GCS cells. The data also demonstrate that downregulation of GRP78 reduces expression of CD44 in a manner similar to that produced by PBI-05204. This suggests that this may be one of the mechanisms associated with regulation of GRP78 in stemness of GSCs. Furthermore, as shown in Fig. 3F, the data show that PBI-05204 also disrupts the binding of GRP78 with CD44 which is associated with inhibition of stemness[71].

Based on the observations of GSC morphology after PBI-05204 treatment, we investigated likely mechanisms of action of PBI-05204 and its involvement in the cell death pathway of glioblastoma stem cells. Receptor interacting protein Kinase 1 (RIPK1) is activated first by phosphorylation, which in turn activates RIPK3 through its kinase activity and the RIPK1/RIPK3 complex is then formed which leads to necroptosis[72,73]. This distinct signature programmed cell death pathway does not involve Caspase-8[[74], [75], [76], [77]] an established marker of apoptosis. As shown in Fig. 4A, PBI-05204 activates RIPK1 phosphorylation followed by RIPK3 phosphorylation and MLKL phosphorylation and formation of the RIPK1-RIPK3 complex which, in turn, primes necroptotic cell death in GSCs[34]. The inhibition of RIPK1 and RIPK3 phosphorylation, alongside the disruption of RIPK1-RIPK3 complex formation due to the RIPK1 inhibitor Necrostatin 1(Nec1) (Fig. 4C), indicates that the GSC cell death induced by PBI-05204 occurs via the RIPK1-RIPK3-MLKL necroptotic pathway. Given that GRP78 is a master regulator for ER stress due to its role as a major ER chaperone and its ability to control the activation of UPR signaling[78,79], we explored whether PBI-05204 also affects regulation of UPR, inositol-required protein 1 (IRE1) and PKR-like endoplasmic reticulum kinase (PERK) as well as the downstream target XBP1 splicing in GBM9 cells. Even though we did observed a moderately higher expression of pIRE1 with a high concentration of PBI-05204 treated GBM9 GSCs, there were no significant changes in the phosphorylation of IRE1 in the presence of a lower concentration of PBI-05204 treatment in comparison to the control. Also, we did not detect XBP1 splicing, a downstream effector of IRE1. At this point we cannot explain the slight increase in the IRE1 phosphorylation with higher concentrations of PBI-05204 treatment, though there was no further activation of this pathway. These findings indicate that PBI-05204 induced necroptosis has a distinct mode of pathway choice through RIPK1-RIPK3 in GSCs and is not likely mediated through regulation of the ER stress pathway.

Xu Yue et al, 2006 have shown that RIPK1 and TRAF2 are required for mitochondrial membrane depolarization during N-methyl-N′- nitro-N-nitrosoguanidine (MNNG)-induced cell death by necroptosis[80]. Necrosome complex formation encompasses ROS generation and subsequent necroptosis[[81], [82], [83], [84]]. These findings indicate that the changes in mitochondrial morphology and possible function could be associated with necroptosis. Necroptotic cell death is characterized by swollen cytoplasmic organelles such as endoplasmic reticulum (ER). The changes in mitochondrial morphology accompanied by rupture of plasma membranes as reflected in Fig. 5C strongly suggest that PBI-05204 produces a necroptotic mediated cell death. Receptor-interacting serine/threonine kinase 1(RIPK1) is a multifunctional signal kinase which resides at intersections of inflammation, immunity, cell stress, cell survival, and cell death. RIPkinase acts as a regulator of FASLG-induced necroptosis in T cells[85]. Our data suggest that GRP78 is a master regulator of stemness in GSCs and that inhibition of GRP78 expression by silencing RNA of GRP78 triggers RIPK1 phosphorylation with subsequent GSC necroptotic cell death.

PBI-05204 has previously been shown to contain a number of important bioactive molecules including oleandrin, oleanolic acid, betulinic acid, and ursolic acid[86]. Of these, oleandrin is known to suppress the proliferation of various tumor types, including GBM by altering membrane fluidity and induction of apoptosis[[87], [88], [89]]. In this study, oleandrin has been shown to inhibit the renewal of GSCs in a concentration- dependent manner. It appears that oleandrin downregulates both Gli1 and NANOG while only having a moderate inhibitory effect on GRP78 which differs slightly from PBI-05204. This suggests that different mechanisms may be involved in antistemness activities of oleandrin and PBI-05204 in GSCs. Additionally, we observed that when PBI-05204 was used with a similar concentration of oleandrin it exerted a stronger inhibitory activity on sphere formation than oleandrin alone in TS453 GSCs, indicating that other components in PBI-05204 may contribute to its suppressive activity on stem cell renewal. Considering our previous findings demonstrating oleandrin's neuroprotective effects via induction of BDNF and Nrf2 antioxidant elements[45,86], our data further supports the notion of anticancer advantages of PBI-05204 in treatment of GBM.

**Fig. 7:**
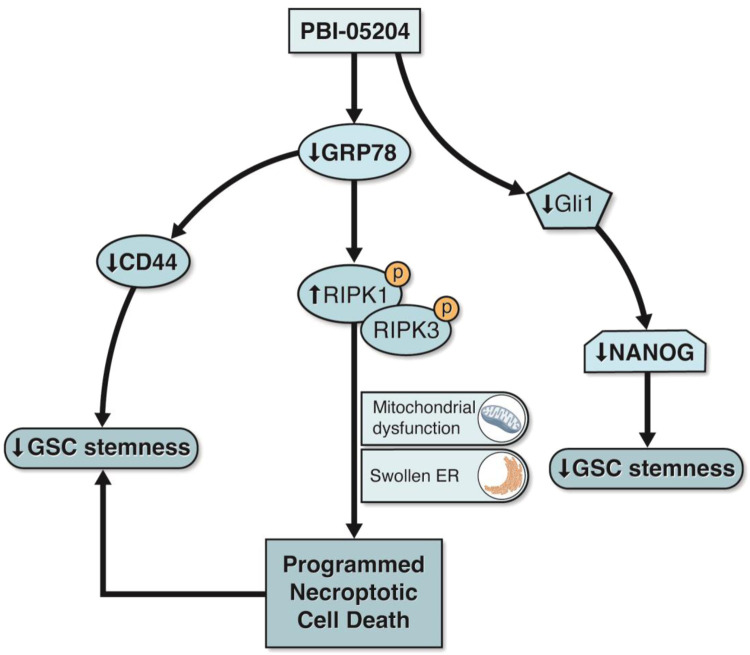
Schematic diagram of the plausible mechanisms of action for the inhibitory activity of PBI-05204 in GSCs.

This investigation has provided several lines of evidence to support the therapeutic potential of PBI-05204 as an innovative approach to affect GBM and GSC survival. The data demonstrate that PBI-05204 inhibits the self-renewal capacity of four patient derived GSCs lines as shown by reductions in sphere formation and cell viability. Tumor size reduction in the PBI-05204 treated xenograft mouse model supports the in vivo ability of this plant derived extract to inhibit self-renewal capacity and viability of GSCs. PBI-05204 has been shown to downregulate the expression of glioblastoma stem cell markers such as CD44, SOX2, Gli1 and NANOG which correlate with the results of inhibition of stem cell renewal capacity and viability as well as tumor volume reduction upon PBI-05204 treatment. In addition, GRP78, an important factor for maintenance of stemness and self-renewal capacity in GSC cell lines, is shown to be inhibited by PBI-05204 and this was confirmed by GRP78 siRNA transfection. PBI-05024 also disrupted binding of GRP78 with CD44 which led to the inhibition of stemness. PBI-05204 upregulates the necroptotic program cell death markers RIPK1 and RIPK3 and enhanced their binding capacity which appears to be mediated by GRP78. Ultrastructural evidence of PBI-05204 treated GSCs include enlarged ER, condensed and elongated mitochondria, and broken cell membranes all of which are hallmarks of necroptosis. Finally, oleandrin, a key molecular component of PBI-05204, was shown to suppress the self-renewal capacity of GCSs and downregulated stem cell marker expression. Based on these findings, we propose a likely series of events that leads to PBI-05204 mediated necroptotic cell death in GSCs as shown in Fig. 7. PBI-05204 inhibits the stemness and growth of GSCs by downregulating GRP78 protein which then leads, in turn, to decreased expression of CD44 and binding of GRP78 and CD44.

Given the pivotal role of GSCs in GBM development, progression, and treatment resistance, coupled with the successful testing of PBI-05204 in phase I and II clinical trials, this botanical drug, either alone or in combination with standard chemotherapy and/or radiotherapy, may offer a more effective way to treat patients with GBM. Its demonstrated ability to inhibit GBM-derived stem cells, penetrate the blood-brain barrier effectively, together with recent evidence supporting its ability to enhance the efficacy of radiotherapy and standard chemotherapy drugs like temozolomide, suggest that PBI-05204 may be a viable candidate for innovative treatment in refractory GBM patients.

## CRediT authorship contribution statement

**Sharmistha Chakraborty:** Writing – review & editing, Writing – original draft, Methodology, Investigation, Data curation. **Daoyan Wei:** Writing – review & editing, Supervision, Investigation, Conceptualization. **Megan Tran:** Project administration, Investigation, Data curation. **Frederick F Lang:** Writing – review & editing, Resources, Project administration, Conceptualization. **Robert A Newman:** Writing – review & editing, Supervision, Project administration, Funding acquisition. **Peiying Yang:** Writing – review & editing, Writing – original draft, Supervision, Project administration, Investigation, Funding acquisition, Conceptualization.

## Declaration of competing interest

R. A. Newman and P. Yang are consultants for Phoenix Biotechnology, Inc. The other authors disclosed no potential conflict of interest.

## Appendix Supplementary materials

Image, application 1
