# Supplementary material for: PBI-05204, a supercritical CO2 extract of Nerium oleander, suppresses glioblastoma stem cells by inhibiting GRP78 and inducing programmed necroptotic cell death
Source: Neoplasia. 2024 May 31;54:101008. doi: 10.1016/j.neo.2024.101008 (PMC11177059; doi:10.1016/j.neo.2024.101008)
Supplement: Supplementary file 1 [file mmc1.pdf]

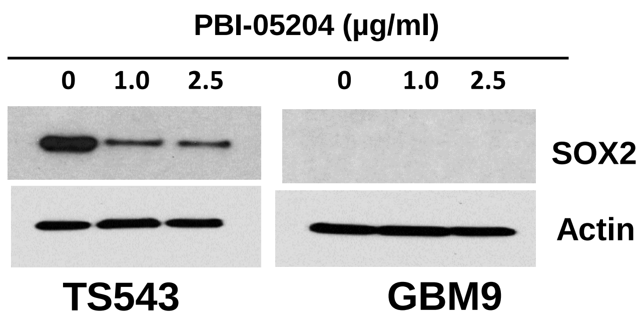

Supplemental Figure 1. Western blot analysis of SOX2 protein in TS543 and GBM9 cells exposed to PBI-05204 at indicated concentrations for 24 hr.

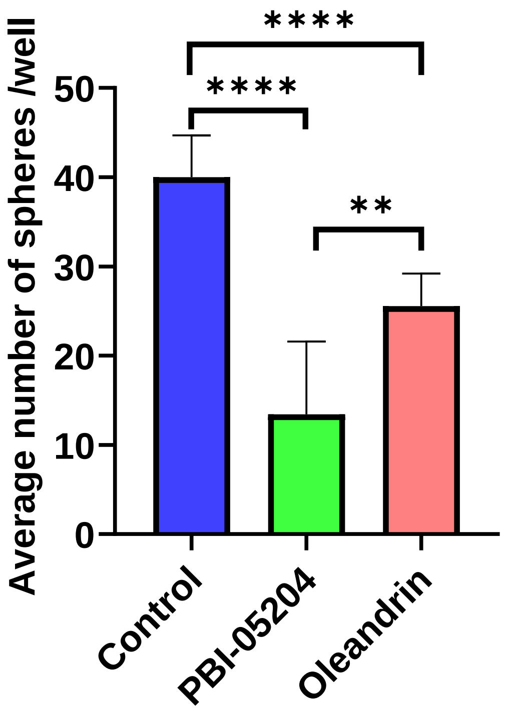

Supplemental Figure 2. The average number of spheroids formed in TS543 GSCs treated with PBI-05204 (1 μg/ml) and oleandrin at the similar concentration presented in PBI-05204 (1 μg/ml). Data are presented as mean ± SD. ** $p < 0.01$; **** $p < 0.0001$.
